# Development of the pulmonary fibrosis, pulmonary vascular resistance, six minute walk distance, B-type natriuretic peptide, age (PVD-B65) risk score for patients with chronic lung disease and pulmonary hypertension

**DOI:** 10.1186/s12890-025-03538-8

**Published:** 2025-02-08

**Authors:** Shameek Gayen, Jay Pescatore, Matthew Bittner, Mario Naranjo, Gerard J. Criner, Sheila Weaver, Temple Lung Center Pulmonary Vascular Disease Working Group, Shameek Gayen, Mario Naranjo, Sheila Weaver, Gilbert D’Alonzo, Lijo Illipparambil, Parth Rali, Seyedmohammad Pourshahid

**Affiliations:** Department of Thoracic Medicine and Surgery, Lewis Katz School of Medicine at Temple University Hospital, Philadelphia, PA 19140 USA

**Keywords:** Chronic lung disease, Pulmonary hypertension, Pulmonary fibrosis, Pulmonary vascular resistance, BNP, Mortality

## Abstract

**Background:**

Pulmonary hypertension (PH) confers increased mortality in patients with chronic lung disease, yet there remains a lack of validated risk assessment tools to prognosticate these patients. We aimed to create a risk assessment tool to stratify patients with chronic lung disease and PH by risk of one-year mortality from time of PH diagnosis.

**Methods:**

This was a retrospective cohort study of patients with chronic lung disease and PH. We identified predictors of one-year mortality via multivariable Cox regression and assigned point values to the identified predictors based on their hazard ratios to comprise the risk score. Patients were stratified into low, intermediate, and high-risk based on total scores. Kaplan–Meier survival analysis comparing the stratified groups was performed. Internal statistical validation was performed via Cox regression with bootstrapping.

**Results:**

The identified predictors of one-year mortality that comprised our risk assessment tool were pulmonary fibrosis without emphysema, pulmonary vascular resistance > 5 WU, six-minute walk distance < 150 m, BNP > 200 pg/mL, and age > 65 years (PVD-B65). Once patients were stratified into the three risk groups, Kaplan–Meier survival analysis demonstrated significant differences in one-year survival between the subgroups (logrank *p* = 0.002). The risk assessment model demonstrated internal validation via bootstrapping (*p* < 0.05).

**Conclusion:**

The PVD-B65 risk assessment tool is a novel, internally validated one-year mortality risk calculator for patients with chronic lung disease and PH that encompasses factors related to pulmonary parenchymal and vascular remodeling. It may help risk stratify and guide therapeutic interventions in patients with chronic lung disease and PH.

**Supplementary Information:**

The online version contains supplementary material available at 10.1186/s12890-025-03538-8.

## Introduction

Pulmonary hypertension (PH) often accompanies chronic lung diseases and is associated with worse outcomes, including reduced functional ability, worsened hypoxemia, and increased risk of mortality [1]. This increased risk of mortality associated with PH is seen across a variety of chronic lung diseases, such as chronic obstructive pulmonary disease (COPD), interstitial lung disease (ILD) including idiopathic pulmonary fibrosis (IPF), and sarcoidosis [2–5]. More recent studies have identified elevated pulmonary vascular resistance (PVR) greater than 5 WU as a predictor of mortality in these patients [6–9].

Several risk assessment tools for mortality have been developed for group 1 pulmonary arterial hypertension (PAH) [10–12]. These risk assessment tools have been found to effectively predict one-year survival and have been validated in patients with PAH based on several disease-specific characteristics, such as six-minute walk distance (6MWD), B-type natriuretic peptide (BNP), functional class (FC), and pulmonary hemodynamics. However, despite the known associated increased risk of mortality of PH and the success of risk assessment development in group 1 PAH, effectively prognosticating patients with chronic lung disease and PH remains challenging. Effective prognostication is necessary as studies have demonstrated increased mortality among patients with chronic lung disease and PH compared to those with group 1 PAH [13, 14].

In this study, we sought to develop and validate a risk assessment tool to predict one-year mortality in patients with chronic lung disease and PH on the basis of factors related to both the underlying lung disease and PH severity.

## Methods

### Study design and definitions

This was a retrospective cohort study of patients with chronic lung disease and PH diagnosed via right heart catheterization (RHC) from 2011 to 2023 at our institution. Those without chronic lung disease, namely group 1 PAH, isolated post-capillary PH due to left-sided heart disease, and chronic thromboembolic PH, were excluded. Chronic lung disease was diagnosed with pulmonary function testing (PFT) and/or computed tomography (CT). Chronic lung disease diagnoses in the cohort included COPD, pulmonary fibrosis without emphysema, non-fibrotic ILD, pulmonary sarcoidosis, and combined pulmonary fibrosis with emphysema (CPFE). The pulmonary fibrosis without emphysema diagnosis included patients with IPF and progressive pulmonary fibrosis in other ILDs. The ILDs in both the pulmonary fibrosis without emphysema diagnosis group and the non-fibrotic ILD group included patients with connective tissue disease (CTD)-ILD, chronic hypersensitivity pneumonitis, and other idiopathic interstitial pneumonias. Subjects with CTD-ILD had significant parenchymal involvement; none had PH as the sole pulmonary manifestation of their connective tissue disease. Subjects with sarcoidosis all had significant parenchymal involvement. PH was defined as resting mean pulmonary artery pressure (mPAP) > 20 mmHg via RHC, which was performed on baseline resting oxygen requirements. New severe PH was classified as PVR > 5 WU in accordance with the 2022 ESC/ERS guidelines [15]. Old severe PH was classified as mPAP > 35 mmHg or mPAP ≥ 25 mmHg with reduced cardiac index in accordance with the 2015 guidelines [16]. Patient demographics, comorbidities, and clinical characteristics —including underlying lung disease, RHC values, PFT data, 6MWD, diffusing capacity of the lungs for carbon monoxide (DL_CO_), oxygen requirement with exertion, and BNP — within three months of PH diagnosis via RHC were collected. The primary outcome was one-year mortality without lung transplantation from diagnosis of PH. The study was approved by the local ethics committee of Temple University (IRB Protocol #31795) and was carried out in accordance with the Transparent reporting of a multivariable prediction model for individual prognosis or diagnosis (TRIPOD) guidelines and checklist [17]. The need for patient consent to participate was waived by the Temple University IRB/ethics committee given the retrospective nature of the study. Procedures were followed in accordance with the ethical standards of the Western IRB and the Helsinki Declaration of 1975.

### Risk assessment development and statistical analysis

All continuous variables were presented as the mean ± standard deviation unless stated otherwise. Categorical variables were compared using the Pearson chi-squared test or Fisher exact test where applicable. Continuous variables were compared between groups using the paired t-test. Univariable Cox regression with subsequent multivariable Cox regression was performed to identify independent and significant predictors of one-year mortality. Missing data for each variable was included in the analysis and displayed if significant. Once predictors of one-year mortality were identified, points were assigned to each variable predictor based on their hazard ratios in the multivariable Cox regression. Risk scores were then calculated for each patient in the cohort and divided into low-risk, intermediate-risk, and high-risk groups for one-year mortality; risk subgroups were determined from the mean and standard deviation of the calculated risk scores. Survival was assessed using the Kaplan–Meier method to test the efficacy of the risk score calculator in prognosticating one-year survival probability. Internal statistical validation of the risk score in predicting one-year mortality was performed via Cox regression with bootstrapping with 1000 samples of the cohort and 95% bias-corrected and accelerated confidence intervals. The statistical analysis was performed using IBM SPSS Statistics, Version 29. A *p*-value < 0.05 was considered statistically significant.

## Results

We identified 793 patients with chronic lung disease and PH. Of the underlying lung diseases, 330 patients had COPD, 251 patients had pulmonary fibrosis without emphysema, 37 patients had non-fibrotic ILD, 70 patients had sarcoidosis with pulmonary involvement, and 105 patients had CPFE. The average age in the cohort was 63.3 years. 403 (50.8%) patients were male, and 390 (49.2%) patients were female (Table 1).

**Table 1:** Demographics and baseline characteristics, *n* = 793

**Age, mean (SD)**	63.3 (9.9)
**Body Mass Index, mean (SD)**	29.1 (7.1)
**Gender**	
Male, *n* (%)	403 (50.8)
Female, *n* (%)	390 (49.2)
**Race/Ethnicity**	
Non-Hispanic Black, *n* (%)	287 (36.2)
Non-Hispanic White, *n* (%)	396 (49.9)
Other, *n* (%)	110 (13.9)
**Underlying Lung Disease**	
COPD, *n* (%)	330 (41.6)
Pulmonary fibrosis without emphysema, *n* (%)	251 (31.7)
Non-Fibrotic ILD, *n* (%)	37 (4.7)
Advanced Pulmonary Sarcoidosis, *n* (%)	70 (8.8)
CPFE, *n* (%)	105 (13.2)

*COPD* Chronic obstructive pulmonary disease, *CPFE* Combined pulmonary fibrosis and emphysema, *ILD* Interstitial lung disease

RHC values diagnosing PH and PFT data available closest to the time of RHC were collected (Table 2). 273 patients met the 2015 ESC/ERS definition of severe PH, while 248 patients met the new 2022 ESC/ERS definition of severe PH, or PVR > 5 WU [15, 16].

**Table 2:** Clinical Characteristics

**RHC Values**	
Right Atrial Pressure, mean (SD)	7.2 mmHg (4.7)
Pulmonary Artery Systolic Pressure, mean (SD)	48.8 mmHg (16.7)
Mean Pulmonary Artery Pressure, mean (SD)	31.2 mmHg (10.1)
Pulmonary Capillary Wedge Pressure, mean (SD)	12.5 mmHg (10.7)
Cardiac Output, mean (SD)	5.0 L/Min (2.7)
Cardiac Index, mean (SD)	2.6 L/Min/m^2^ (0.7)
Pulmonary Vascular Resistance, mean (SD)	5.0 WU (4.0)
Old Pre-Capillary PH, *n* (%)	429 (54)
New Pre-Capillary PH, *n* (%)	575 (73)
Old Severe PH, *n* (%)	273 (34)
New Severe PH, *n* (%)	248 (31)
PCWP > 15 mmHg, *n* (%)	113 (14.2)
**PFT Values**	
% Predicted FEV1, mean (SD)	47% (24.1)
% Predicted FEV1 > 80%, *n* (%)	79 (10.0)
% Predicted FEV1 50–80%, *n* (%)	277 (34.9)
% Predicted FEV1 30–50%, *n* (%)	211 (26.6)
% Predicted FEV1 < 30%, *n* (%)	189 (23.8)
Missing, *n* (%)	37 (4.7)
% Predicted FVC, mean (SD)	60.1% (23.3)
% Predicted FVC > 80%, *n* (%)	152 (19.1)
% Predicted FVC 40–80%, *n* (%)	521 (65.7)
% Predicted FVC < 40%, *n* (%)	83 (10.5)
Missing, *n* (%)	37 (4.7)
% Predicted DLCO, mean (SD)	28.1% (15.2)
DLCO < 40%, *n* (%)	407 (51.3)
6 Minute Walk Distance, mean (SD)	239.3 m (96.2)
> 300 m, *n* (%)	93 (11.7)
150—300 m, *n* (%)	479 (60.4)
< 150 m, *n* (%)	93 (11.7)
Missing, *n* (%)	128 (16.2)
Oxygen Requirement on Exertion, mean (SD)	5.5 L/min (4.9)
Oxygen requirement > 5 L/min, *n* (%)	404 (50.9)
BNP	450.2 pg/mL (368.15)
BNP ≤ 200 pg/mL	221 (27.9)
BNP > 200 pg/mL, *n* (%)	184 (23.2)
Missing, *n* (%)	388 (48.9)

*BNP* B-type natriuretic peptide, *DLCO* Diffusing capacity of the lung for carbon monoxide, *FEV1* Forced expiratory volume in 1 s, *FVC* Functional vital capacity, *PFT* Pulmonary function test

### Predictors of one-year mortality

The mean time to one-year outcome was 8.4 months; 55 patients (6.9%) died within one-year of PH diagnosis via RHC. Univariate Cox regression was performed to identify variables associated with one-year mortality prior to potential lung transplantation, with variables showing significant association (*p* < 0.05) subsequently utilized in multivariable Cox regression to determine independent and significant predictors of one-year mortality (Table 3). Underlying lung disease, age > 65, old criteria for severe PH, new criteria for severe PH (PVR > 5 WU), b-type natriuretic peptide (BNP) > 200 pg/mL, forced expiratory volume in 1 s (FEV1), functional vital capacity (FVC), and 6MWD were significantly associated with one-year mortality in univariate analysis and utilized in multivariable analysis. Pulmonary fibrosis without emphysema (HR 4.42, 95% CI 1.41–13.87, *p* = 0.01), PVR > 5 WU (HR 2.17, 95% CI 1.53–3.57, *p* = 0.03), 6MWD < 150 m (HR 2.50, 95% CI 1.41–8.45, *p* = 0.04), BNP > 200 pg/dL (HR 2.77, 95% CI 1.18–6.51, *p* = 0.02), and age > 65 years (HR 3.30, 95% CI 1.36–8.02, *p* = 0.01) were identified to be significant and independent predictors of one-year mortality. Among patients with pulmonary fibrosis without emphysema, IPF (HR 4.49, 95% CI 1.29–15.68, *p* = 0.02) and other fibrotic ILD (HR 4.36, 95% CI 1.24–15.25, *p* = 0.02) had similar association with one-year mortality.

**Table 3:** Predictors of one-year mortality

Variable	Univariate Cox Regression	Multivariable Cox Regression
Lung Disease (COPD as reference)^a^		
Pulmonary fibrosis without emphysema^b^	HR 2.43, 95% CI 1.20–4.93, ***p* = 0.01**	HR 4.42, 95% CI 1.41–13.87, ***p* = 0.01**
Non-fibrotic ILD	HR 1.04, 95% CI 0.24–4.50, *p* = 0.96	HR 0.69, 95% CI 0.08–6.33, *p* = 0.74
Sarcoidosis	HR 1.59, 95% CI 0.63–4.05, *p* = 0.33	HR 2.81, 95% CI 0.74–10.74, *p* = 0.13
CPFE	HR 1.61, 95% CI 0.67–3.88, p = 0.29	HR 1.78, 95% CI 0.33–9.79, *p* = 0.51
Age > 65^a,b^	HR 1.87, 95% CI 1.09–3.22, ***p* = 0.02**	HR 3.30, 95% CI 1.36–8.02, ***p* = 0.01**
Male gender	HR 1.51, 95% CI 0.75–1.98, *p* = 0.35	
RAP	HR 1.00, 95% CI 0.94–1.05, *p* = 0.90	
PCWP	HR 1.00, 95% CI 0.97–1.02, *p* = 0.92	
CI < 2.5	HR 0.72, 95% CI 0.42–1.22, *p* = 0.22	
Pre-capillary PH (old criteria)	HR 1.27, 95% CI 0.74–2.18, *p* = 0.38	
Pre-capillary PH (new criteria)	HR 0.88, 95% CI 0.50–1.56, *p* = 0.66	
Severe PH (old criteria)^a^	HR 2.26, 95% CI 1.33–3.84, ***p* = 0.003**	HR 1.17, 95% CI 0.53–2.57, *p* = 0.70
Severe PH (new criteria)^a,b^	HR 2.50, 95% CI 1.48–4.25, ***p* < 0.001**	HR 2.17, 95% CI 1.53–3.57, ***p* = 0.03**
Systolic Blood Pressure	HR 1.00, 95% CI 0.99–1.02, *p* = 0.50	
Heart Rate	HR 1.01, 95% CI 0.99–1.02, *p* = 0.42	
BMI	HR 0.95, 95% CI 0.91–1.01, *p* = 0.10	
BNP > 200^a,b^	HR 3.03, 95% CI 1.59–5.75, ***p* < 0.001**	HR 2.77, 95% CI 1.18–6.51, ***p* = 0.02**
FEV1 (> 80% predicted as reference)^a^		
50–80% predicted	HR 0.37, 95% CI 0.16–0.84, ***p* = 0.02**	HR 0.97, 95% CI 0.28–3.32, *p* = 0.96
30–50% predicted	HR 0.63, 95% CI 0.29–1.39, *p* = 0.25	HR 1.21, 95% CI 0.29–5.08, *p* = 0.80
< 30% predicted	HR 0.36, 95% CI 0.15–0.88, ***p* = 0.03**	HR 2.00, 95% 0.30–13.20, *p* = 0.47
FVC (> 80% predicted as reference)^a^		
40–80% predicted	HR 0.48, 95% CI 0.26–0.88, ***p* = 0.02**	HR 0.40, 95% CI 0.13–1.18, *p* = 0.10
< 40% predicted	HR 0.93, 95% CI 0.38–2.25, *p* = 0.87	HR 1.28, 95% CI 0.28–5.83, *p* = 0.75
DLCO < 40% predicted	HR 0.69, 95% CI 0.32–1.49, *p* = 0.35	
6MWD (> 300 m as reference)^a^		
150–300 m	HR 1.27, 95% CI 0.54–2.98, *p* = 0.59	HR 2.15, 95% CI 0.68–6.78, *p* = 0.19
< 150 meters^b^	HR 3.14, 95% CI 1.27–7.79, ***p* = 0.01**	HR 2.50, 95% CI 1.41–8.45, ***p* = 0.04**
Oxygen > 5L/min	HR 1.74, 95% CI 0.95–3.20, *p* = 0.07	

^a^utilized in multivariable Cox regression to determine significant and independent predictors of mortality

^b^significant and independently associated with mortality and utilized in creation of one-year mortality risk score

Multicollinearity analysis was performed among the variables identified as independent and significant predictors (Supplemental Table 1). All predictors (lung disease, age > 65 years, PVR > 5 WU, BNP > 200 pg/dL, and 6MWD) had a variance inflation factor (VIF) close to 1, suggesting mild correlation between them in the multivariable Cox regression model, but not to a degree that impacts the reliability of the model.

### Risk assessment score development

The following points were assigned to the identified predictors of one-year mortality based on their respective hazard ratios in the multivariable Cox regression analysis to comprise the risk assessment score (Table 4): 4 points for pulmonary fibrosis without emphysema, 3 points for age > 65 years, 2 points for PVR > 5 WU, 3 points for BNP > 200 pg/dL, and 2 points for 6MWD < 150 m. The risk score ranges from 0 to 14. There were 407 patients in our cohort with full data for whom a risk score could be calculated. The mean risk score was 4.6, with a standard deviation of 2.9; two standard deviations from the mean were used to identify lower and upper limits for the risk groups. Patients were characterized as low-risk for scores 0 through 3 (155 patients), intermediate-risk for scores 4 through 10 (212 patients), and high-risk for scores 11 through 14 (40 patients).

**Table 4:** PVD-B65 Risk Assessment Score Calculator Breakdown

Variable	Points
**P**ulmonary fibrosis without emphysema	+ 4
P**V**R > 5 WU	+ 2
6MW**D** < 150 m	+ 2
**B**NP > 200 pg/dL	+ 3
Age > **65** years	+ 3
**Risk Score Groups**	
Maximum Score	14 points
Low-Risk	0 to 3 points (155 patients)
Intermediate-Risk	4 to 10 points (212 patients)
High-Risk	11 to 14 points (40 patients)

*6MWD* 6-min walk distance, *BNP* B-type natriuretic peptide, *PVR* Pulmonary vascular resistance, *WU* Woods units

### Survival analysis by risk score severity

**Fig. 1:**
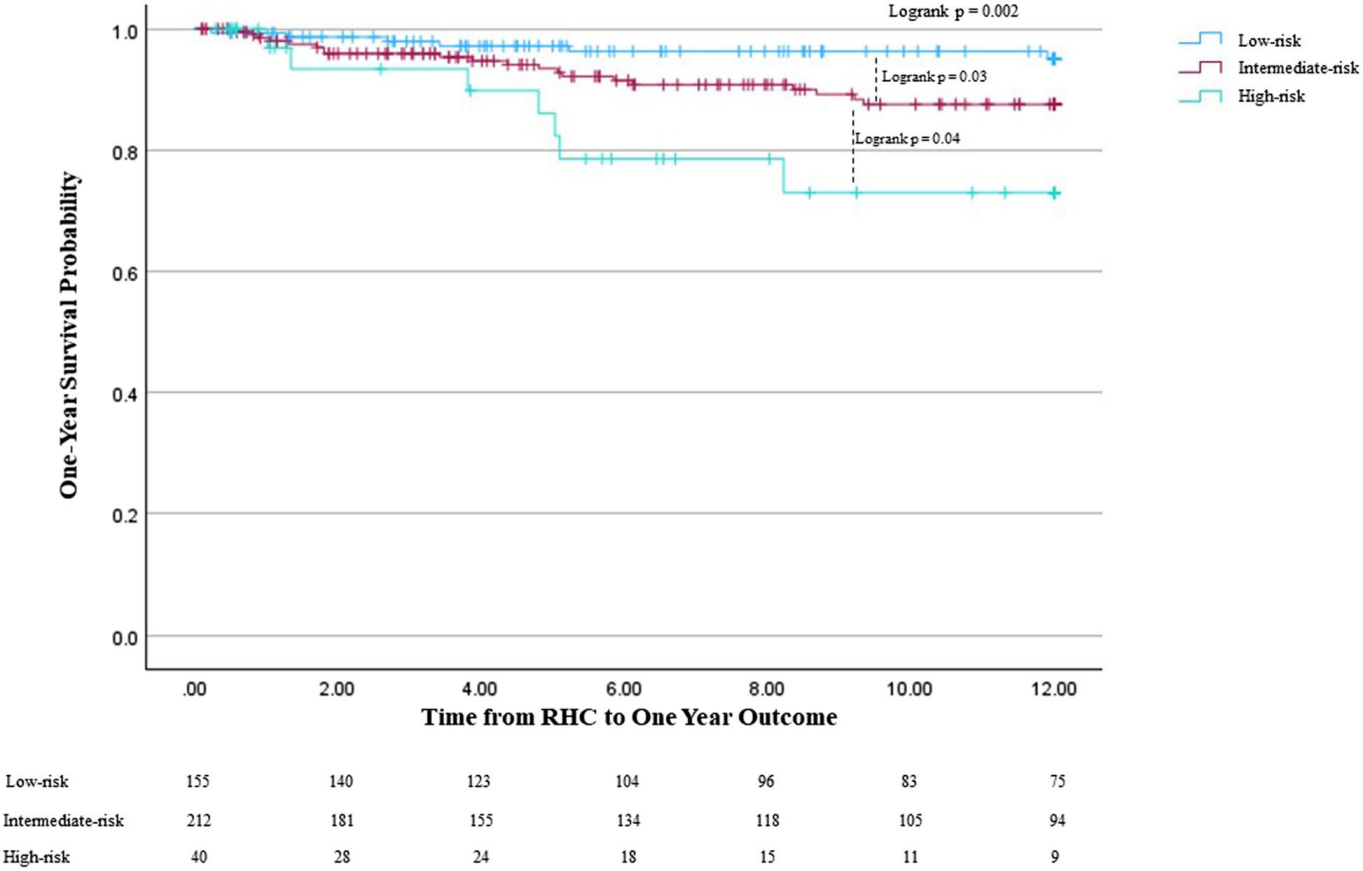
Kaplan Meier One-Year Survival Analysis Comparing Stratified Risk Groups. 1 = low risk, 2 = intermediate risk, 3 = high risk. Kaplan Meier analysis comparing the three stratified risk groups based on the PVD-B65 tool. The number of patients at risk is displayed at the bottom of the graph. Changes in each graph due to death before potential lung transplant while also censoring those who received lung transplant, as they would no longer be at risk of death before transplant. Lung transplantation is not reflected in the actual Kaplan–Meier survival curves. RHC: right heart catheterization

One-year mortality rates were: 3.9% (6 patients) in the low-risk group, 9.4% (20 patients) in the intermediate-risk group, and 17.5% (7 patients) in the high-risk group. Survival analysis to compare the risk score severity groups was performed using the Kaplan–Meier method (Fig. 1) and Cox regression. There was a significant difference in one-year survival probability among the groups (logrank *p* = 0.002), with the intermediate-risk and high-risk groups demonstrating worse one-year survival probability. The intermediate-risk group had a lower one-year survival probability than the low-risk group (logrank *p* = 0.03), and the high-risk group had lower one-year survival probability than the intermediate-risk group (logrank *p* = 0.04).

Cox regression analyzing the association between the risk assessment score severity groups and one-year mortality demonstrated that compared to the low-risk group, the intermediate-risk group was significantly associated with increased risk of one-year mortality (HR 2.59, 95% CI 1.04–4.65, *p* = 0.04). The high-risk group was significantly associated with an increased risk of one-year mortality as compared to the low-risk group as well (HR 6.20, 95% CI 2.08–18.48, *p* = 0.001).

### Internal validation of risk assessment score calculator

We performed internal validation of our risk assessment score via Cox regression with bootstrapping. In the bootstrap analysis of 1000 cohort subsets, both the intermediate-risk (*p* = 0.03) and the high-risk (*p* < 0.001) groups were significantly associated with one-year mortality risk as compared to the low-risk group, similar to the original Cox regression findings for our risk score calculator. These findings demonstrate the internal statistical validity of our risk score calculator in predicting one-year mortality among cohorts of patients with chronic lung disease and PH.

## Discussion

We developed a risk assessment score that effectively stratifies patients with chronic lung disease and PH by their risk of one-year mortality from time of PH diagnosis and was internally validated via bootstrapping. The PVD-B65 risk assessment tool was developed through analysis of multiple prognostic factors related to age as well as to both underlying lung disease and PH severity, namely pulmonary fibrosis without emphysema, elevated PVR, decreased 6MWD, and elevated BNP. Notably, our risk assessment tool was effectively utilized across a wide spectrum of underlying lung diseases.

Difficulty prognosticating and subsequently stratifying patients with chronic lung disease and PH is likely due to the numerous factors that influence outcomes in these patients; both lung disease and PH severity play roles, as well as non-disease specific characteristics such as age. An earlier study of this same cohort identified underlying lung disease diagnosis, reduced lung function, elevated PVR, and reduced 6MWD all to be independent and significant predictors of overall mortality [18]. The PVD-B65 risk assessment tool effectively captures both lung disease severity and PH severity when prognosticating patients with chronic lung disease and PH.

Pulmonary fibrosis without emphysema was the strongest predictor of one-year mortality and accordingly given the highest weight in the risk assessment calculation. With regards to ILD, IPF is characterized by early mortality, while the presence and higher degrees of fibrosis are associated with increased mortality in other ILDs, such as CTD-ILD [19]. CPFE has a higher risk of mortality than COPD or emphysema alone [20]. Among chronic lung disease and PH, IPF is known to correlate independently with increased mortality among patients with parenchymal lung disease and concomitant PH, while those with COPD and PH have improved survival [21, 22]. Patients with IPF and concomitant emphysema, or CPFE, have been found to have worse outcomes than those with IPF alone, which is at least in part associated with the development of severe PH [23]. Interestingly, we did not find CPFE to be a predictor of one-year mortality in our analysis, though we did find it to predict overall mortality in our earlier study of this cohort [18]. It may be that one year is too short a time to capture the mortality risk associated with CPFE, whereas IPF is known to have an aggressive, early mortality rate; one study found the median survival of patients with pulmonary fibrosis and advanced emphysema was 62 months compared to 29 months in patients with pulmonary fibrosis without emphysema [24]. One of the salient and most unique features of our score is that clinicians can include any fibrotic lung disease to the PVD-B65 score. Both IPF and other fibrotic ILD had nearly identical risks of one-year mortality, illustrating that IPF is not the only driving factor in the predictive value of pulmonary fibrosis without emphysema on one-year mortality. We were able to capture and appropriately weight the risk of mortality attributed to the diagnosis of pulmonary fibrosis without emphysema in our risk assessment tool.

While pulmonary fibrosis is predictive of one-year mortality in our cohort, other markers of pulmonary parenchymal severity such as FVC and oxygen requirement were not. This may be due to the presence of PH in these patients, with the severity of pulmonary vascular disease and its associated markers (i.e. elevated PVR, BNP, 6MWD, etc.) potentially driving one-year mortality to a greater extent.

The evidence for elevated PVR in predicting mortality is compelling and led to a shift in defining severe PH in chronic lung disease away from mPAP-based criteria to PVR greater than 5 WU in the 2022 ESC/ERS guidelines for the diagnosis and management of PH [15]. This was driven by studies demonstrating that this PVR cutoff better predicted mortality in patients with ILD-PH and COPD-PH [6, 7]. PVR of 5 WU or greater is also associated with decreased transplant-free survival in patients with sarcoidosis-associated PH (SAPH) [9]. Severe PH, now defined as PVR greater than 5 WU, has demonstrated strong prognostic efficacy across PH with various lung diseases; this was demonstrated in our cohort for both one year mortality in this study and overall mortality [18]. Notably, the older, mPAP-driven definition of severe PH was not a predictor of mortality in our cohort; this is again reflective of the growing evidence that PVR is a better prognostic marker in chronic lung disease and PH than mPAP [6, 7]. Additionally, PVR less than 5 WU is a protective factor in the REVEAL 2.0 risk score for PAH [10]. It is not surprising that a PVR greater than 5 WU was an independent and significant predictor of one-year mortality in this cohort and is an integral factor in the PVD-B65 risk assessment tool. Notably, neither the 2015 nor 2022 criteria for pre-capillary PH were predictive of one-year mortality in this cohort. Given the severity of PH as well as the high proportion of pre-capillary PH in the cohort, this is not surprising.

The 6-min walk test is a valuable clinical tool, reflecting patient functional status, pulmonary hemodynamics, and lung function. Patient 6MWD, notably at a reduced value, is predictive of outcomes across several lung diseases with or without associated PH, such as COPD, IPF, and sarcoidosis [25–27]. Reduced 6MWD also predicts outcomes in group 1 PAH and is included in PAH risk score calculators, such as REVEAL 2.0 [10, 28]. Thus, the association of 6MWD less than 150 m with one-year mortality, while not surprising, is significant. It reflects the severity of pulmonary vascular disease, the burden of underlying lung disease, and functional status of the patients in our cohort; as such, incorporating 6MWD in our one-year mortality risk assessment tool is essential as a value that encompasses both PH and lung disease severity.

Plasma BNP levels correlate with right ventricle (RV) dysfunction in PH, with elevated levels found in RV pressure overload due to PH, positive correlation with mPAP and right atrial pressure, and negative correlation with cardiac output [29]. Elevated BNP levels as well as the trajectory of BNP have been identified as predictors of mortality in patients with PAH [30]. Unsurprisingly, BNP is an important component of the REVEAL 2.0 risk score for PAH [10]. Given the severity of PH in our cohort of patients with chronic lung disease and PH (mean PVR was 5.0 WU), it is unsurprising that elevated BNP was a significant and independent predictor of one-year mortality and an important component of our risk assessment tool.

Age is a previously validated variable associated with increased risk of mortality in patients with Group 1 PAH, as seen in the REVEAL registry data [31]. The COMPERA registry demonstrated that higher age was an independent predictor of death among patients with ILD-PH, while other studies have demonstrated increased age to be predictive of mortality across other lung diseases with PH as well [3, 6]. Whether this reflects worsening disease with advanced age or a separate process contributing to mortality risk is unclear, but our risk assessment tool does capture and incorporate the associated mortality risk with increased age.

The PVD-B65 risk assessment score is a novel tool in that it encompasses and can be applied to a wide range of lung diseases, including COPD, pulmonary fibrosis of various etiologies, and sarcoidosis. Prior attempts to prognosticate outcomes in similar patients include applying validated group 1 PAH risk scores to patients with lung disease and PH. A truncated version of the ESC/ERS risk stratification model for PAH was applied to patients with ILD and severe PH, demonstrating prognostic relevance. However, the variables used to risk stratify these ILD-PH patients included 6MWD and those related to PH severity alone (i.e. BNP, right atrial pressure, cardiac index, etc.); no ILD-specific variables were included [32]. There have been some risk scores developed for ILD-PH specifically. Utilizing the patients in the INCREASE trial evaluating inhaled Treprostinil, two risk calculators, non-invasive and invasive, were developed and predicted clinical worsening over the 16-week study period [33]. While these authors utilized FVC in their calculator, this was the only lung related variable considered, and their risk calculator was limited to 16 weeks and a more limited scope of underlying lung disease. A second tool designed to assess severity in ILD-PH patients and guide therapeutic decisions utilized functional class, pulmonary hemodynamics, and echocardiogram findings, but again did not include any variables reflective of the underlying lung disease, such as the presence of pulmonary fibrosis [34]. Our risk assessment tool accounts for the significance of pulmonary fibrosis in the outcomes of patients with chronic lung disease and PH in addition to PH severity and was developed by analyzing patients with a wide spectrum of underlying lung disease.

There are several limitations to this retrospective study. Unlike randomized trials, the pertinent variables were not collected at protocolized, scheduled visits. As such, not all patients had all the data available when determining predictors of mortality and assigning risk assessment scores. However, we did include patients with missing data in the analysis of one-year mortality, making the model and risk score broadly generalizable. Our cohort encompasses many patients referred for lung transplant evaluation, suggesting a possible higher degree of illness severity with regards to their pulmonary parenchymal and vascular disease than the general population of chronic lung disease and PH patients. However, our identified predictors of one-year mortality applied in the risk assessment tool are factors that have been identified and validated as predictors of mortality in numerous studies involving patients with lung disease and patients with pulmonary hypertension, while encompassing a potentially “sicker” patient population. Additionally, this risk score requires external validation, which is an important future direction.

## Conclusion

The PVD-B65 risk assessment tool is a novel tool for prognostication in patients with chronic lung disease and PH. By identifying predictors of one-year mortality with exhaustive Cox regression analysis, we composed a risk assessment tool that captures the multifaceted and complex nature of factors that affect outcomes in patients with lung disease and PH. We identified and utilized in one, comprehensive score variables that reflect parenchymal severity, pulmonary vascular severity, functional status, and age, all of which have been identified in the literature as predictive of or associated with mortality among patients with lung disease and/or PH. The PVD-B65 risk assessment tool for one-year mortality was developed from and successfully internally validated within a large cohort of patients with chronic lung disease and PH, consisting of COPD-PH, ILD-PH, SAPH, and CPFE-PH. While external validation of our risk assessment tool is necessary, the PVD-B65 risk score can help aid in prognosticating patients diagnosed with lung disease and PH and, in turn, help guide therapeutic decisions, such as pulmonary vasodilator treatment regimens and referrals to lung transplantation centers for evaluation. Earlier identification of higher risk patients and initiation of appropriate interventions may help reduce the morbidity and mortality associated with this serious yet underrecognized condition.

## Supplementary Information

Supplementary Material 1.

## Data Availability

The data that support the findings of this study are available from the corresponding author upon reasonable request.
