# Supplementary material for: Development of the pulmonary fibrosis, pulmonary vascular resistance, six minute walk distance, B-type natriuretic peptide, age (PVD-B65) risk score for patients with chronic lung disease and pulmonary hypertension
Source: BMC Pulm Med. 2025 Feb 8;25:69. doi: 10.1186/s12890-025-03538-8 (PMC11807298; doi:10.1186/s12890-025-03538-8)
Supplement: Supplementary file 1 — Supplementary Material 1. [file 12890_2025_3538_MOESM1_ESM.docx]

**Supplemental Table 1: Multicollinearity Analysis**

| **Predictor** | **Variance Inflation Factor** |
| --- | --- |
| Lung Disease | 1.05 |
| Age > 65 years | 1.12 |
| PVR > 5 WU | 1.07 |
| BNP > 200 pg/mL | 1.07 |
| 6MWD | 1.07 |

6MWD: 6-minute walk distance

BNP: B-type natriuretic peptide

WU: Woods units
